# Estrogen Protects the Female Heart from Ischemia/Reperfusion Injury through Manganese Superoxide Dismutase Phosphorylation by Mitochondrial p38β at Threonine 79 and Serine 106

**DOI:** 10.1371/journal.pone.0167761

**Published:** 2016-12-08

**Authors:** Tao Luo, Han Liu, Jin Kyung Kim

**Affiliations:** Division of Cardiology, Department of Medicine, School of Medicine, University of California Irvine, Irvine, California, United States of America; University of PECS Medical School, HUNGARY

## Abstract

A collective body of evidence indicates that estrogen protects the heart from myocardial ischemia/reperfusion (I/R) injury, but the underlying mechanism remains incompletely understood. We have previously delineated a novel mechanism of how 17β-estradiol (E2) protects cultured neonatal rat cardiomyocytes from hypoxia/reoxygenation (H/R) by identifying a functionally active mitochondrial pool of p38β and E2-driven upregulation of manganese superoxide dismutase (MnSOD) activity via p38β, leading to the suppression of reactive oxygen species (ROS) and apoptosis. Here we investigate these cytoprotective actions of E2 *in vivo*. Left coronary artery ligation and reperfusion was used to produce I/R injury in ovariectomized (OVX) female mice and in estrogen receptor (ER) null female mice. E2 treatment in OVX mice reduced the left ventricular infarct size accompanied by increased activity of mitochondrial p38β and MnSOD. I/R-induced infarct size in ERα knockout (ERKO), ERβ knockout (BERKO) and ERα and β double knockout (DERKO) female mice was larger than that in wild type (WT) mice, with little difference among ERKO, BERKO, and DERKO. Loss of both ERα and ERβ led to reduced activity of mitochondrial p38β and MnSOD at baseline and after I/R. The physical interaction between mitochondrial p38β and MnSOD in the heart was detected by co-immunoprecipitation (co-IP). Threonine 79 (T79) and serine 106 (S106) of MnSOD were identified to be phosphorylated by p38β in kinase assays. Overexpression of WT MnSOD in cardiomyocytes reduced ROS generation during H/R, while point mutation of T79 and S106 of MnSOD to alanine abolished its antioxidative function. We conclude that the protective effects of E2 and ER against cardiac I/R injury involve the regulation of MnSOD via posttranslational modification of the dismutase by p38β.

## Introduction

The incidence of cardiovascular diseases (CVD) is significantly lower in women than in age-matched men until menopause, after which CVD risk accelerates to equal or exceed that in men. Female sex hormone, estrogen, is thought to play a central role in gender differences apparent in CVD, and this theory has been tested in many controlled preclinical experiments [1–5] and clinical trials [6–8]. Overall, a cumulative body of work published to date suggests that endogenous estrogen has a potent impact on the heart, conferring generally protective effects in a variety of pathological states, such as in ischemia.

Numerous studies support the contribution of reactive oxygen species (ROS) in the pathogenesis of myocardial ischemia/reperfusion (I/R) injury, leading to cardiac apoptosis and ventricular dysfunction [9]. Estrogen is well-known to suppress oxidative stress in the heart and vasculature [10–12]. The hormone’s antioxidative effect may play a significant role in protecting cardiomyocytes against I/R injury, though the detailed mechanism of how estrogen suppresses oxidative stress is not fully understood. The hormone’s signaling to nitric oxide synthase (NOS) and thioredoxin in the heart has been proposed [13, 14]. Furthermore, in our previous work, we showed that 17β-estradiol (E2) inhibited ROS generation *in vitro* in a simulated I/R in the form of hypoxia/reoxygenation (H/R) applied to cultured neonatal rat cardiomyocytes (NRCM) [15, 16]. This protection was dependent on both estrogen receptor alpha (ERα) and beta (ERβ). The suppression of mitochondrial ROS production was associated with upregulating the activity of p38β and prevented cardiomyocyte apoptosis. We further delineated the mechanism by which E2-induced p38β regulates redox by demonstrating a pool of functionally active mitochondrial p38β and the kinase-substrate relationship between p38β and manganese superoxide dismutase (MnSOD) [17].

In the present study, we report mechanistic details of E2 actions in protecting the heart from myocardial I/R *in vivo*, using a comprehensive approach of whole animal models and molecular analyses. Our findings correlate the E2-mediated cardioprotection to the interaction between mitochondrial p38β and MnSOD in the heart, in which the phosphorylation of the MnSOD amino acid residues threonine 79 (T79) and serine 106 (S106) by the kinase is essential to ROS suppression. We also performed a direct comparison between the role of ERα and ERβ in cardioprotection by employing a whole animal model of I/R, using wild type (WT), ERα knockout (ERKO), ERβ knockout (BERKO), and ER α and β double knockout (DERKO) female mice, and report that both ER subtypes are necessary for cardioprotection during I/R stress.

## Materials and Methods

### Animals

The investigation conformed to the Guide for the Care and Use of Laboratory Animals published by the US National Institutes of Health (NIH Publication No. 85–23, revised 1996). All study was approved by the ethical committee for animal experiments at University of California Irvine (Protocol # 2007–2755).

Ovariectomized (OVX), 6 week-old, female WT mice were purchased from Harlan Laboratories. The animals were housed in light/dark (12hr-12hr circle) for 2 weeks. E2 (0.1 mg/pellet, 21-day release; Innovative Research of America) or placebo pellet was administered subcutaneously 7 days before the surgery. Then, the OVX mice with or without E2 underwent cardiac surgery to simulate cardiac I/R injury, as detailed in the next section.

Female WT C57BL6 mice and ER null mice (25–30 g) were also used. Male and female double heterozygous (ERα^+/-^β^+/-^) mice with a mixed C57BL6 background were obtained from the laboratory of Dr. Kenneth Korach and were bred at our animal facility to produce WT [ERα^+/+^β^+/+^], ERKO [ERα^-/-^β^+/+^], BERKO [ERα^+/+^β^-/-^] and DERKO [ERα^-/-^β^-/-^] offspring [18]. Genotyping of tail DNA was performed to screen the target mice using polymerase chain reaction (PCR). The details of screening were presented in S1 Fig. After genotyping, the mice were individually housed per the institutional animal protocol.

### Myocardial I/R injury

The myocardial I/R injury in a whole animal model was created as described previously [19]. Briefly, mice were anesthetized with a mixture of xylazine (5 mg/kg) and ketamine (100 mg/kg) administered intraperitoneally. The mice were then intubated and ventilated using a mouse ventilator (Type 845, Harvard Apparatus) with 120 breaths per min and a stroke volume of 300 μL. A left thoracotomy was performed, and the left coronary artery (LCA) was ligated for 45 min at the level of the tip of the left auricle, followed by reperfusion by release of the ligation. The chest was closed, and mice were allowed to recover for the following 24 hr before the harvest of the heart. Sham groups were operated in parallel, but without the LCA ligation.

### 2,3,5-Triphenyl-2H-tetrazolium chloride (TTC) staining

At the end of 24 hr reperfusion, the mice were sacrificed under anesthesia, and the hearts were removed and immediately frozen. The frozen hearts were sectioned at the papillary muscle level. The myocardial slices were incubated in 2% TTC buffer in 37°C for 30 min. After staining, the TTC buffer was replaced, and the slices were fixed with 4% formaldehyde for measurement of the infarcted areas using NIH ImageJ. The infarct size was calculated and presented as the percentage of the cross-sectional area of the left ventricle (LV).

### Mitochondria isolation from the heart

Twenty four hours after reperfusion, mice were sacrificed under anesthesia, and the hearts were removed quickly and placed on ice. The left ventricular tissue (~200 mg) 2 mm below the cross section of ligature point was obtained. A commercially available mitochondria isolation kit was used for cardiac mitochondria isolation (Thermo Scientific). The purified mitochondrial pellets were dissolved in 300 μl TBS containing 2% 3-[(3-cholamidopropyl) dimethyl-ammonio]-1-propanesulfonate (CHAPS). Protein concentration was quantified by bicinchoninic acid BCA Protein Assay (Pierce). The samples were stored at −80°C for a maximum of 1 week before use.

### Co-immunoprecipitation (Co-IP)

p38β kinase and MnSOD were precipitated from mitochondrial lysate by adding anti-p38β or anti-MnSOD antibodies. After incubation for 2 hr at 4°C, 10 μl of protein A-Sepharose beads (Invitrogen) were added, and incubated with moderate stirring at 4°C for 1 hr. Nonspecific IgG and the whole heart tissue homogenate were also used as negative and positive control, respectively. The beads were washed, and the immunoprecipitated protein complex was loaded onto 12% SDS-PAGE gel, electrophoresed and processed for immunoblotting. To determine a molecular interaction between p38β and MnSOD, the p38β-immunoprecipitating complex was blotted for MnSOD, and MnSOD-immunoprecipitating complex blotted for p38β.

### Neonatal rat cardiomyocyte isolation and culture

NRCM were isolated from 1–3 day old Sprague-Dawley rats according to the protocol previously described [17]. The harvested cells were then incubated in DMEM/F12 cell culture medium (Gibco) supplemented with 10% fetal bovine serum (Invitrogen) and 1% antibiotic-antifungal mixture (Sigma) at 37°C before use.

### Hypoxia/reoxygenation

H/R was induced in a hypoxia chamber according to the protocol previously described [17]. Briefly, NRCM were placed overnight at 37°C in an air-tight hypoxia chamber (Billups-Rothenbery) filled with 95% N2, 4% CO2, and 1% O2 to yield less than 2% oxygen, measured by an oxygen analyzer (Vascular Technology), followed by 2 hr reoxygenation by exposure to room air at 37°C.

### Immunofluorescence (IF) staining

After fixation and permeabilization, NRCM (1×10^4^ cells/mL) or female heart tissue sections (in 5 μm thickness) were stained with anti-p38β primary antibody (sc-6187-R, Santa Cruz) at room temperature for 1 hr. Following triple wash in PBS, they were incubated with Fluorescein anti-rabbit IgG (V0729, Vector laboratories) for 30 min in dark, rinsed in PBS three times, then co-incubated with 1 mM MitoTracker deep red 633 (Molecular Probes) to stain mitochondria. Heart tissue slices were mounted with mounting medium containing 4’,6-diamidino-2-phenylindole (DAPI), and observed under confocal microscope (Zeiss) for p38β (green fluorescence), mitochondria (red) and nucleus (blue).

### Western blotting

Immunoblotting was performed using the following primary antibodies: anti-phospho-p38 (sc-17852-R, Santa Cruz), anti-p38β (sc-6187-R, Santa Cruz), anti-β-actin (sc-130656, Santa Cruz), anti-MnSOD (SC-30080, Santa Cruz), and anti-COX IV (#4844S, Cell Signaling Technology). To detect phosphorylated p38β (p-p38β) in mitochondria, anti-p-p38 antibody was first conjugated to protein A-Sepharose beads to immunoprecipitate all phosphorylated p38 kinase isoforms from mitochondrial lysate. The p-p38 complex was then blotted for p38β to yield p-p38β. Immunoreactive bands were visualized by the enhanced chemiluminescence method (Pierce ECL 2 Western Blotting Substrate) and quantified by NIH ImageJ software.

### MnSOD activity assay

Mitochondrial pellets were isolated as described above and re-suspended in mitochondrial cold buffer (20 mM HEPES, pH 7.2, 1 Mm EGTA, 210 mM mannitol, and 70 mM sucrose). The mitochondrial MnSOD activity was then measured with a superoxide dismutase kit (Cayman Chemical), in the presence of 2 mM potassium cyanide to inhibit both the Cu/Zn-SOD and extracellular SOD activity, according to the manufacturer’s protocol.

### MnSOD peptides synthesis and MnSOD point mutation

The amino acid sequences of human MnSOD (protein accession: CAA32502.1) was analyzed for high surface probability, protein flexibility and kinase activity score, as determined by proprietary bioinformatics programs for the peptide analysis (Life Tein). Thus, we identified 3 regions spanning amino acid residue 16–35, 61–80, and 91–110, containing candidate serine or threonine residues. The segment spanning the amino acid residues 181–200 had a high kinase activity score but no serine or threonine, and served as a negative control. These four peptide sequences were synthesized. Each peptide was then subjected to in-vitro kinase assays with purified p38β, as described below. The information of the synthesized peptides is presented in the supplementary data (S2A Fig and S1 Table).

pcDNA 3.1/ (WT) MnSOD 3’-flags plasmid and pcDNA3.1/ (Mut) MnSOD 3’-flags plasmid were gifts from Dr. Jianjian Li at the University of California, Davis. The sequence information of the plasmids is presented in the supplementary data (S3 Fig). The mutant MnSOD plasmid, pcDNA3.1/ (Mut) MnSOD 3’-flags, contains a change of amino acid residue 106 from serine to alanine (S106A). We created a new point mutation to replace the amino acid 79 from threonine to alanine (T79A), using the QuikChange II Site-Directed Mutagenesis Kit (Catalog # 200523, Agilent Technologies) and the WT MnSOD plasmid provided above, after the residue was also found to be phosphorylated by p38β (S3 Fig). NRCM were transfected with the WT and two mutant MnSOD plasmids by Lipofectamine 3000 (Invitrogen, R705-07) for 2 days to overexpress WT or mutant MnSOD. The MnSOD protein was purified by ANTI-FLAG M2 Affinity Gel (Sigma-Aldrich) and stored at -80°C for further experiment.

### Kinase activity assay

Radioactive and non-radioactive methods were used to measure the kinase activity. The purified activated p38β kinase (Sigma) was used to test phosphorylation of the MnSOD peptides and purified full-length WT or mutant MnSOD proteins. The purified activating transcription factor 2 (ATF2)-fusion protein (Cell Signaling Technology) was used as the positive control substrate [20].

In the radioactive method, adenosine triphosphate (ATP) labeled with ^32^P (ATP, [γ-^32^P]- 6000Ci/mmol 10mCi/ml Lead, 250 μCi, Perkin Elmer) was incubated with purified activated p38β kinase and substrate of interest at 30°C for 30 min, as previously described [17]. The samples were loaded and electrophoresed on a SDS-PAGE gel. The gel was then fixed, dried and exposed on film at -80°C overnight, and the film was developed the following day to image the radiolabeled substrates.

In the non-radioactive method, an ADPsensor Universal Kinase Activity Assay Kit (BioVision) was used per manufacturer’s instructions. Purified WT and mutant MnSOD served as the substrates of p38β (100 ng). At the end of 120 min of incubation, the kinase activity of p38β was measured by fluorescence spectrophotometer (Ex/Em = 535/587 nm). The sample kinase activity was then calculated according to the formula per manufacturer’s instruction.

### ROS detection assay

The WT and mutant MnSOD plasmids described above were transfected into NRCM for 2 days followed by H/R. Subsequent ROS production in NRCM was measured by ROS-ID^™^ Total ROS Detection Kit per manufacturer’s instruction (Enzo life sciences). One mM MitoTracker deep red 633 (Molecular Probes) was added to co-stain mitochondria in the cells, followed by observation of immunofluorescence under confocal microscope (Zeiss).

### Statistical analysis

All experiments were repeated at least three times. Data were expressed as the mean ± SEM, with *p* < 0.05 to indicate statistical significance. The paired *t*-test was used for comparisons between two groups. One-way ANOVA with post hoc analysis by the Fisher exact probability test was employed for multiple treatment groups. At least 3 animals were used per treatment group for reproducibility. All analyses were performed using SPSS 13.0 software (SPSS Inc.).

## Results

### Estrogen protects the heart against I/R stress by increasing the functionally active pool of p38β

**Fig 1:**
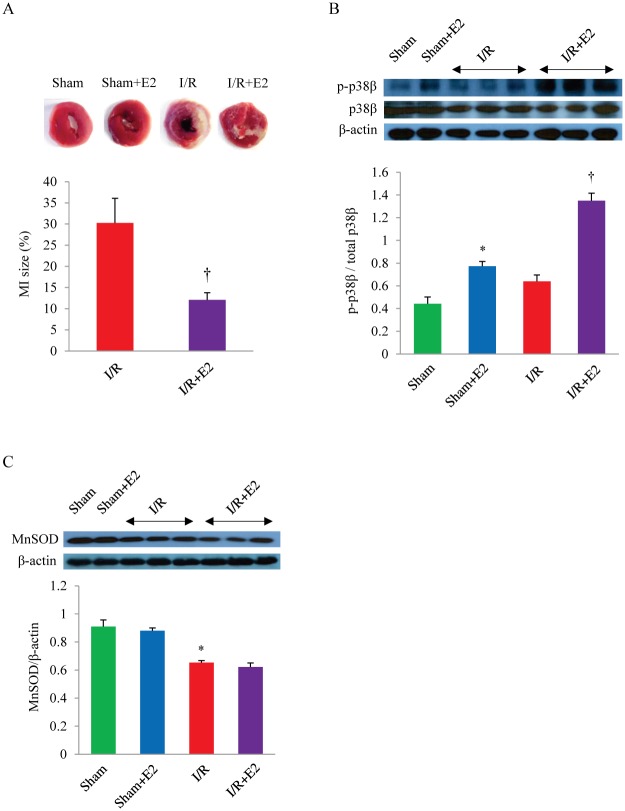
The effect of E2 on myocardial infarct size, p38β activation and MnSOD expression. (A) Representative TTC staining of the heart sections in OVX mice with or without E2 supplementation. (B) The phosphorylation of p38β in the left ventricle of OVX mice with or without E2. (C) The protein level of MnSOD in the left ventricle of OVX mice with or without E2 supplementation.*P<0.05 vs. Sham; ^†^ *P*<0.05 vs. I/R; n = 3 in each group. E2, 17β-estradiol; I/R, ischemia/reperfusion; MnSOD, manganese superoxide dismutase.

We induced myocardial I/R by ligation of LCA for 45 min followed by reperfusion for 24 hr in OVX mice with or without E2 supplementation. The I/R-induced infarct size, determined by TTC staining, in OVX mice without E2 was approximately 30% of the left ventricle. However, the infarct size decreased by half in mice treated with E2 (Fig 1A). The reduced infarct size correlated with E2-induced augmentation of phosphorylated p38β, as evidenced by western blotting of the LV homogenates obtained post sham or I/R surgery for total p38β and activated p38β kinase (p-p38β) (Fig 1B). Of note, because there is no commercially available antibody against p-p38β to be used in immunoblotting, all phosphorylated p38 kinase isoforms (p-p38) was first immunoprecipitated with anti-p-p38 antibody, followed by blotting of the p-p38 complex with p38β-specific antibody to identify p-p38β, as previously described [17]. Estrogen supplementation increased the p-p38β: total p38β ratio in both sham and I/R group, indicating that E2 treatment specifically enhances the activation of the p38β kinase (Fig 1B). The mechanism behind the increase in p-p38β (i.e. activation of the p38β kinase) by E2 has been detailed in our previous work, and involves E2-mediated cross talk with and activation of the upstream PI3K/AKT cytoprotective pathway signaling that augments the activation of p38β and the kinase activity [15]. Given the previously reported relationship between p38β and MnSOD in NRCM [17], we also examined the MnSOD expression in LV and found that the MnSOD protein level was attenuated post I/R (Fig 1C). Estrogen supplementation had no effect on the total protein level of MnSOD in the heart homogenates. This is consistent with prior observations made from an in-vitro H/R model in NRCM that E2 does not alter MnSOD protein expression, while the hormone significantly augments the enzymatic activity [17]. The purported mechanism was activation of MnSOD by E2 via the p38β kinase, which was shown to exist in NRCM mitochondria and phosphorylate MnSOD.

### Estrogen increases the activity of mitochondrial p38β and MnSOD

**Fig 2:**
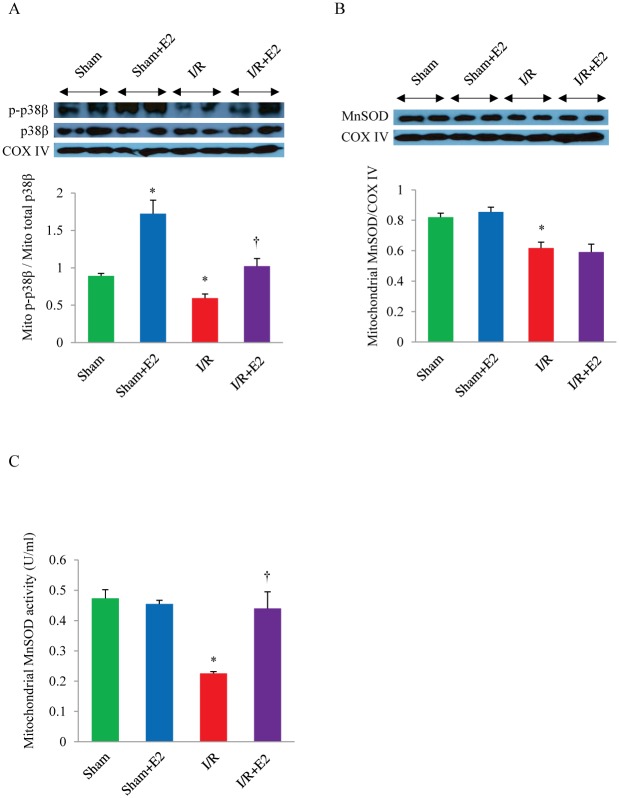
The effect of E2 on the mitochondrial p38β activation and MnSOD activity. (A) Western blots and quantitative analysis of p-p38β and total p38β in mitochondria isolated from the left ventricle of the indicated treatment groups of OVX mice. (B) The protein level of MnSOD in mitochondria isolated from the left ventricle of the indicated treatment groups of OVX mice. (C) The activity of MnSOD in mitochondria isolated from the left ventricle of the indicated treatment groups of OVX mice.*P<0.05 vs. Sham; ^†^ *P*<0.05 vs. I/R; n = 3 in each group. E2, 17β-estradiol; I/R, ischemia/reperfusion; MnSOD, manganese superoxide dismutase, COX IV, cytochrome *c* oxidase subunit IV.

To see if E2 modulation of the p38β kinase-MnSOD relationship holds true *in vivo*, we isolated mitochondria from the left ventricle of OVX mice post I/R injury with or without E2 treatment. In line with the whole cell lysate data above, the mitochondrial pool of p-p38β was increased by E2 treatment in the sham and I/R group (Fig 2A). I/R alone led to decreased p-p38β, whereas addition of E2 (I/R + E2) reversed this attenuation to the baseline level. This finding also correlates with the reduction of infarct size seen in the same treatment group (Fig 1A). It is notable that specifically the ratio of p-p38β over the total p38β in mitochondria was increased by E2, indicating that the hormone treatment leads to augmentation of activated mitochondrial p38β subset.

In order to see if E2-mediated activation of p38β leads to a change in the MnSOD function, we examined the protein level and activity of MnSOD isolated from LV mitochondria (Fig 2B). The total mitochondrial MnSOD protein level decreased after I/R, unaffected by estrogen supplementation. However, as E2 augments the functionally active pool of mitochondrial p38β, shown above, and has also been demonstrated to reduce H/R-induced ROS production in cultured cardiomyocytes, we postulated that E2 might exert its antioxidant effects in the heart by modulating MnSOD via post-translation modification by p38β [15, 17]. Therefore, we measured the MnSOD activity in the left ventricle post sham surgery or I/R injury with or without E2. As shown in Fig 2C, the activity of MnSOD was down-regulated by more than half after myocardial I/R, but almost completely recovered by estrogen supplementation. Together, these data indicate that E2 is able to selectively enhance the activity of mitochondrial p38β associated with preservation of MnSOD activity post I/R.

### Both ERα and ERβ support the activity of p38β and MnSOD and are necessary for cardioprotection from I/R stress

Of the two classical ER subtypes, ERα and ERβ, it is not clear which mediates cardioprotection during ischemia-related injury. Both receptor isoforms are found in the mitochondria of cardiomyocytes and participate in the E2-initiated regulation of mitochondrial function [21–23]. Ex-vivo models that tested either ER subtype separately in its role of cardioprotection from I/R injury led to positive results for each subtype [24, 25]. There has been one study making a head-to-head comparison between ERα and ERβ in this regard, again in an ex-vivo model of global I/R, and it suggests that ERβ may play a larger protective role in the female heart [26].

**Fig 3:**
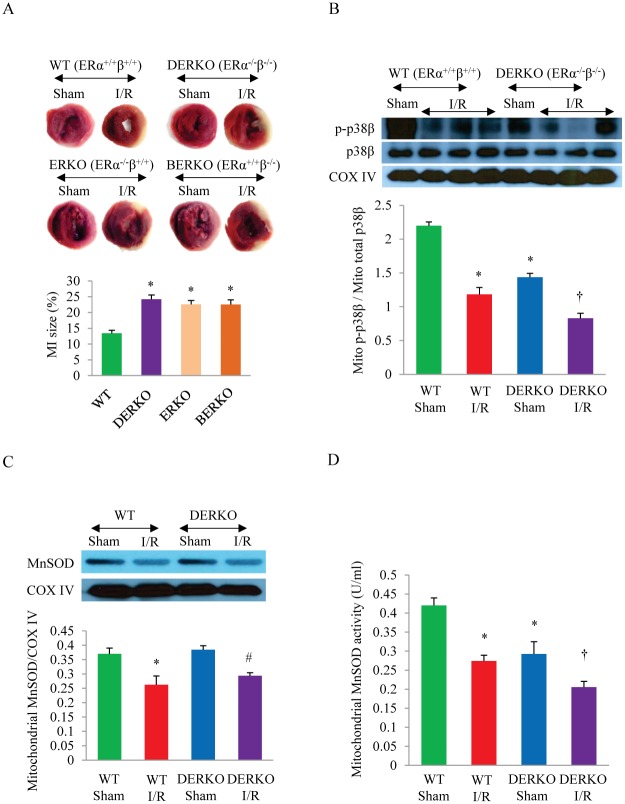
The effect of ER subtypes on the infarct size and the activity of mitochondrial p38β and MnSOD. (A) Representative TTC staining and quantitative analysis of the heart sections in wild type and estrogen receptor knockout mice after myocardial I/R. *P<0.05 vs. WT; n = 3 in each group. (B) Immunoblotting of the active p38β (p-p38β) level in mitochondrial fraction isolated from the left ventricle of WT and DERKO mice after sham or I/R surgery and quantitative analysis of the ratio between p-p38β over total p38β in mitochondria. *P<0.05 vs. WT Sham; ^†^ *P*<0.05 vs. DERKO Sham; n = 3 in each group. (C) The protein level of MnSOD in mitochondrial fraction isolated from the left ventricle of WT and DERKO mice after sham or I/R surgery, shown in immunoblots with quantitative analysis. *P<0.05 vs. WT Sham; ^#^ *P*<0.05 vs. DERKO Sham; n = 3 in each group. (D) The activity of MnSOD in mitochondrial fraction isolated from the left ventricle of WT and DERKO mice after sham or I/R surgery. *P<0.05 vs. WT Sham; ^†^ *P*<0.05 vs. DERKO Sham; n = 3 in each group. I/R, ischemia/reperfusion; MI, myocardial infarction; MnSOD, manganese superoxide dismutase, COX IV, cytochrome c oxidase subunit IV.

To investigate the role of ERα and ERβ in E2 -mediated cardioprotection *in vivo*, we compared the left ventricular infarct size and the mitochondrial p38β and MnSOD activity of WT, ERKO, BERKO, and DERKO female mice subjected to I/R injury by coronary artery ligation and release *in vivo*, as described above. As shown in Fig 3A, the extent of infarct was significantly worse in DERKO mice, compared to WT. Interestingly, while the absolute size of infarct was slightly greater in DERKO, there was no significant statistical difference in the extent of I/R-induced infarct among DERKO, ERKO and BERKO mice. Taken together, these in-vivo data demonstrate that both ERα and ERβ play an essential role in cardioprotection.

We then probed for mitochondrial p-p38β in the left ventricle of WT and DERKO mice to examine the contribution of ER in activating mitochondrial p38β in the heart. Western blotting results showed that mitochondrial p-p38β, the activated form of the kinase, was significantly decreased after I/R in WT mice (Fig 3B). In DERKO mice, the baseline p-p38β level was much lower, compared to that of WT mice, indicating that functional ERs are integral to maintaining p38β activity. The mitochondrial p-p38β level was further reduced in DERKO after I/R, suggesting that unopposed I/R injury is detrimental to p38β activation without the presence of functional ER, and this finding is consistent with a large infarct size seen in DERKO mice post I/R (Fig 3A). I/R stress reduced the protein level of MnSOD in both WT and DERKO hearts (Fig 3C). The absence of ERα and ERβ did not affect the baseline MnSOD expression, as the level of the MnSOD protein did not differ between WT and DERKO mice hearts at baseline. However, the activity of MnSOD was significantly impacted by the deletion of ERα and ERβ (Fig 3D). The baseline MnSOD activity was much lower in DERKO mice hearts, compared with WT. With I/R stress, the MnSOD activity in DERKO was further decreased, significantly lower than WT + I/R. The data suggest that the presence of ERα and ERβ is vital to MnSOD activation needed in defense against I/R injury and cardioprotection *in vivo*, and that this ER-mediated promotion of the dismutase activity likely involves a non-genomic pathway.

### Interaction of p38β and MnSOD in mitochondria

**Fig 4:**
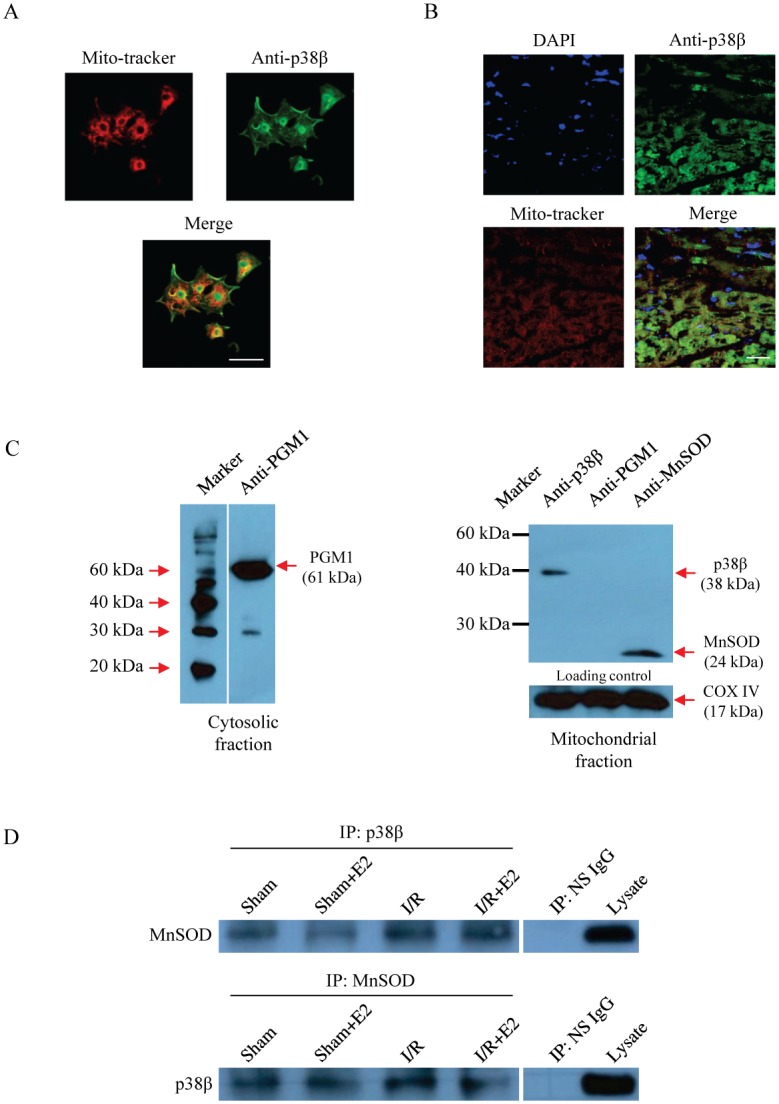
Physical interaction between mitochondrial p38β and MnSOD *in vivo*. (A) Cellular localization of p38β in neonatal rat cardiomyocytes. The white scale bar represents 25 μm. (B) Cellular localization of p38β in the left ventricle of the female mouse heart. The white scale bar represents 25 μm. (C) Immunoblotting of a cytosolic marker, PGM1, in the cytosolic fraction and of p38β and MnSOD in the mitochondrial fraction from OVX female heart, with Cox IV as a mitochondrial marker. (D) Immunoblotting of MnSOD in the p38β-immunoprecipitate and immunoblotting of p38β in the MnSOD-immunoprecipitate from the mitochondrial fractions in the OVX hearts of the indicated treatment groups. NS IgG, nonspecific IgG input. Lysate, unfractionated LV homogenate. DAPI, 4’,6-diamidino-2-phenylindole; PGM1, phosphoglucomutase-1; MnSOD, manganese superoxide dismutase; COX IV, cytochrome c oxidase subunit IV; E2, 17β-estradiol; IP, immunoprecipitation.

In light of the presented data supporting E2-mediated activation of p38β and MnSOD associated with reduced infarct, we sought evidence for the interaction between the p38β kinase and MnSOD in the heart. First, we confirmed the presence of the kinase in mitochondria where MnSOD resides. The immunofluorescent staining of NRCM demonstrated that part of p38β localized to mitochondria and nucleus (Fig 4A). This is consistent with the previous reports of the p38β subcellular localization pattern in cultured cardiac and non-cardiac cells [17, 27, 28]. Similarly, in the whole heart sections of adult female mice, p38β was present in mitochondria (Fig 4B). In addition to the immunofluorescent studies, we confirmed p38β expression in the mitochondria isolated from the left ventricle by immunoblotting. PGM1, a known cytosolic marker, served as a negative control to demonstrate the purity of our mitochondrial extraction from the heart (Fig 4C). Cox IV served as a mitochondrial marker. In the mitochondrial fraction isolated from the whole heart, p38β was identified to be present by immunoblotting, complementing the immunofluorescent data above. We then used co-immunoprecipitation (co-IP) to detect a physical interaction between mitochondrial p38β and MnSOD in the left ventricle treated with or without I/R and/or E2 (Fig 4D). Briefly, the lysate from the ventricle was immunoprecipitated with anti-p38β, then the p38β-containing complex was blotted with anti-MnSOD antibody. The reverse was done to confirm the interaction, whereby the lysate was immunoprecipitated with anti-MnSOD, then the MnSOD-immunocomplex blotted with anti-p38β. While not quantitative in nature to assess the strength or temporality of the interaction, the co-IP study demonstrates clearly that the kinase and MnSOD have a physical association. We have previously shown that p38β phosphorylates MnSOD *in vitro*, and our current in-vivo data demonstrating a direct interaction between the kinase and MnSOD in the heart introduce evidence of a contact expected of an enzyme-substrate relationship.

### p38β phosphorylates T79 and S106 of MnSOD

To further investigate the novel relationship between MnSOD and p38β and to provide mechanistic details behind the E2-derived cardioprotection via p38β, we began to identify the MnSOD residues that might be phosphorylated by the kinase. Since p38β is a serine/threonine kinase that belongs to the mitogen-activated protein kinase (MAPK) superfamily, we screened for serine or threonine residues mostly likely to be phosphorylated by a MAPK, based on surface probability, protein flexibility, and high probability of kinase activity per the peptide analysis by the proprietary bioinformatics program (see Methods). Three such serine- or threonine-containing segments within MnSOD were identified from the 222 amino acid, human MnSOD protein sequence. A fourth segment identified to have high probability of kinase activity did not contain serine or threonine residues, and served as a negative control sequence for p38β kinase activity. We then synthesized four 20-amino acid MnSOD peptides derived from these segments (three containing the candidate serine or threonine residues and one serving as a negative control peptide) (S2A Fig and S1 Table). These peptides were used as substrates in in-vitro kinase assays with p38β.

**Fig 5:**
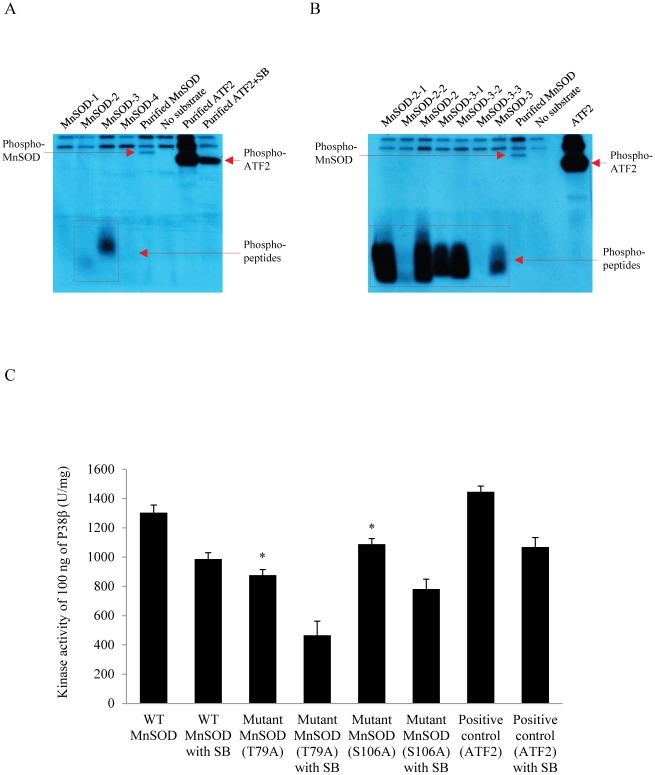
The phosphorylation of MnSOD peptides and mutants T79A and S106A by p38β. (A) Radioactive kinase activity of p38β on wild type MnSOD peptides. MnSOD-1, MnSOD-2, MnSOD-3, MnSOD-4 peptide contains the human WT MnSOD sequence from amino acid position 16–35, 61–80, 91–110, and 181–200, respectively. The dotted box marks the peptides phosphorylated and radiolabeled by p38β. (B) Radioactive kinase activity of p38β on wild type and mutant MnSOD peptides. MnSOD 2–1 had T65 mutated to alanine (T65A). MnSOD-2-2 had T79 changed to alanine (T79A). MnSOD-3-1, MnSOD-3-2 and MnSOD-3-3 contained point mutations S99A, T103A, and S106A, respectively. The dotted box marks the peptides radiolabeled by p38β and those mutants not phosphorylated by the kinase. (C) Kinase activity of p38β on full length wild type and mutant MnSOD, T79A and S106A. *P<0.05 vs. WT MnSOD. MnSOD, manganese superoxide dismutase; ATF2, activating transcription factor 2; SB, SB 203580 (1 μM). 2 μg of purified MnSOD and ATF2 was used, respectively.

Two out of the four peptides, named MnSOD-2 and MnSOD-3, were phosphorylated by active p38β kinase (Fig 5A). Within the MnSOD-2 peptide were two threonine residues corresponding to amino acid position 65 (T65) and 79 (T79) of human MnSOD protein. Within the MnSOD-3 peptide were two serine residues corresponding to amino acid position 99 (S99) and 106 (S106), and a threonine residue corresponding to 103 (T103). In order to see if each of these residues is a potential phosphorylation site by p38β, another set of MnSOD peptides were synthesized, in which the sequences of MnSOD-2 and MnSOD-3 now contained a point mutation of these five serine or threonine residues, changing individual serine or threonine to alanine (S2B Fig and S2 Table). The mutant peptide, MnSOD-2-1, contained a change of T65 to alanine (T65A). MnSOD-2-2 had T79 changed to alanine (T79A). Similarly, peptides named MnSOD-3-1, MnSOD-3-2 and MnSOD-3-3 contained point mutations S99A, T103A, and S106A, respectively. These mutant MnSOD peptides were then subjected to kinase assays to identify the residue(s) likely to be phosphorylated by p38β. Compared to the wild type counterparts, MnSOD-2-2 (T79A) and MnSOD-3-3 (S106A) failed to be phosphorylated by p38β, suggesting that T79 and S106 were the likely residues to be involved in the p38β-mediated phosphorylation (Fig 5B). Of note, the top two bands seen in the kinase reaction represent autophosphorylation of the p38β kinase [29, 30].

To extend these results to the interaction between the kinase and full-length MnSOD in cardiomyocytes, we transfected the full-length WT and mutant MnSOD plasmids into NCRM and performed kinase assays (Fig 5C). The mutants contained either T79A or S106A point mutation (S3 and S4 Figs). The results in Fig 5C showed that phosphorylation of MnSOD T79A and MnSOD S106A by p38β was significantly reduced, compared to WT MnSOD or positive control substrate, ATF2. SB 203580 (SB) was used as a p38β-specific inhibitor. Taken together, these findings indicate that T79 and S106 of MnSOD represent the likely phosphorylation sites by p38β in cardiomyocytes.

### T79 and S106 of MnSOD are important in ROS suppression in cardiomyocytes

**Fig 6:**
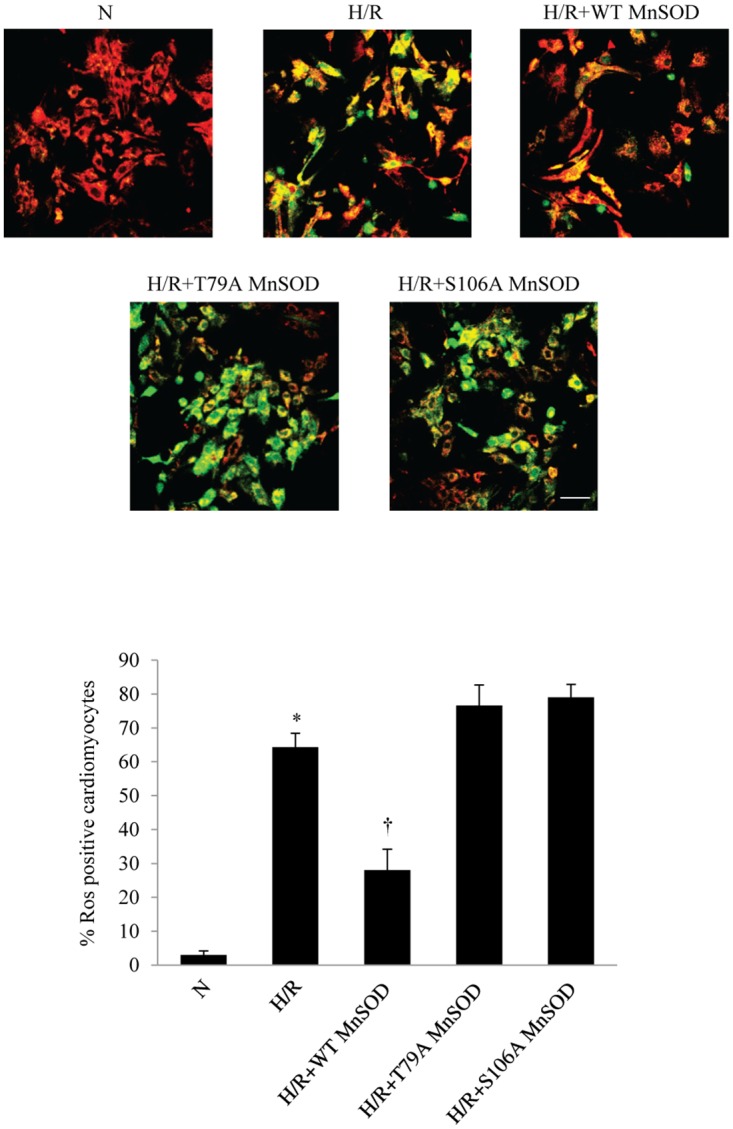
The effect of T79A or S106A mutant MnSOD on ROS generation in cardiomyocytes after H/R. Intracellular ROS (fluorescent green) was detected in NRCM after full length WT, T79A mutant MnSOD, or S106A mutant MnSOD plasmid was transfected. Mitochondria are co-stained with MitoTracker (red). Representative images of cells are shown with quantitative analysis. *P<0.05 vs. N; ^†^ *P*<0.05 vs. H/R. The white scale bar represents 25 μm. N, normoxia; H/R, hypoxia/reoxygenation; MnSOD, manganese superoxide dismutase; ROS, reactive oxygen species.

To test whether the identified MnSOD residues, T79 and S106, hold functional implication in cytoprotection, we tested the effect of WT and the two mutant MnSOD, T79A and S106A, against H/R injury in cardiomyocytes. Our hypothesis was that the two residues phosphorylated by p38β are important in the antioxidative function of MnSOD, thereby essential in protection of cardiomyocytes from oxidative stress. Therefore, we transfected and expressed the WT and the two mutant plasmids, T79A MnSOD and S106A MnSOD, in NRCM. The transfection efficiency of these three plasmids at 2 days was about 30% (S5 Fig). This is in line with a known, relatively low transfection rate of plasmids into primary cell culture of cardiomyocytes [31]. Cardiomyocytes were then exposed to H/R accordingly to the previously described protocol as above. As expected, ROS generation, represented in fluorescent green, was increased after H/R, compared to normoxia (Fig 6). The transfection of WT MnSOD significantly decreased ROS generation. However, transfecting T79A MnSOD or S106A MnSOD mutant into the cells abrogated the ability of MnSOD to attenuate intracellular ROS production post H/R. The results indicate that the two amino acids, T79 and S106, are critical to the core function of MnSOD as a major regulator of oxidative stress in cardiomyocytes. Posttranslational modification of MnSOD by p38β kinase, thus, represents an integral part of cytoprotective mechanisms mediated by the E2-p38β signaling.

## Discussion

The female sex hormone, 17β-estradiol, and its receptors confer various cardioprotective effects in the heart, including regulation of cardiac metabolism, attenuation of cardiomyocyte apoptosis, promotion of cardiac regeneration, modulation of myocardial hypertrophy, and calibration of electromechanical coupling and arrhythmogenicity (reviewed in [32]). We have previously reported the ability of E2 to protect cardiomyocytes from hypoxia-driven apoptosis by activation of p38β and downregulation of mitochondrial ROS production [15]. We also demonstrated a pool of mitochondrial p38β and the novel kinase-substrate relationship between p38β and MnSOD as part of the E2-mediated cytoprotection from ROS in NRCM [17].

**Fig 7:**
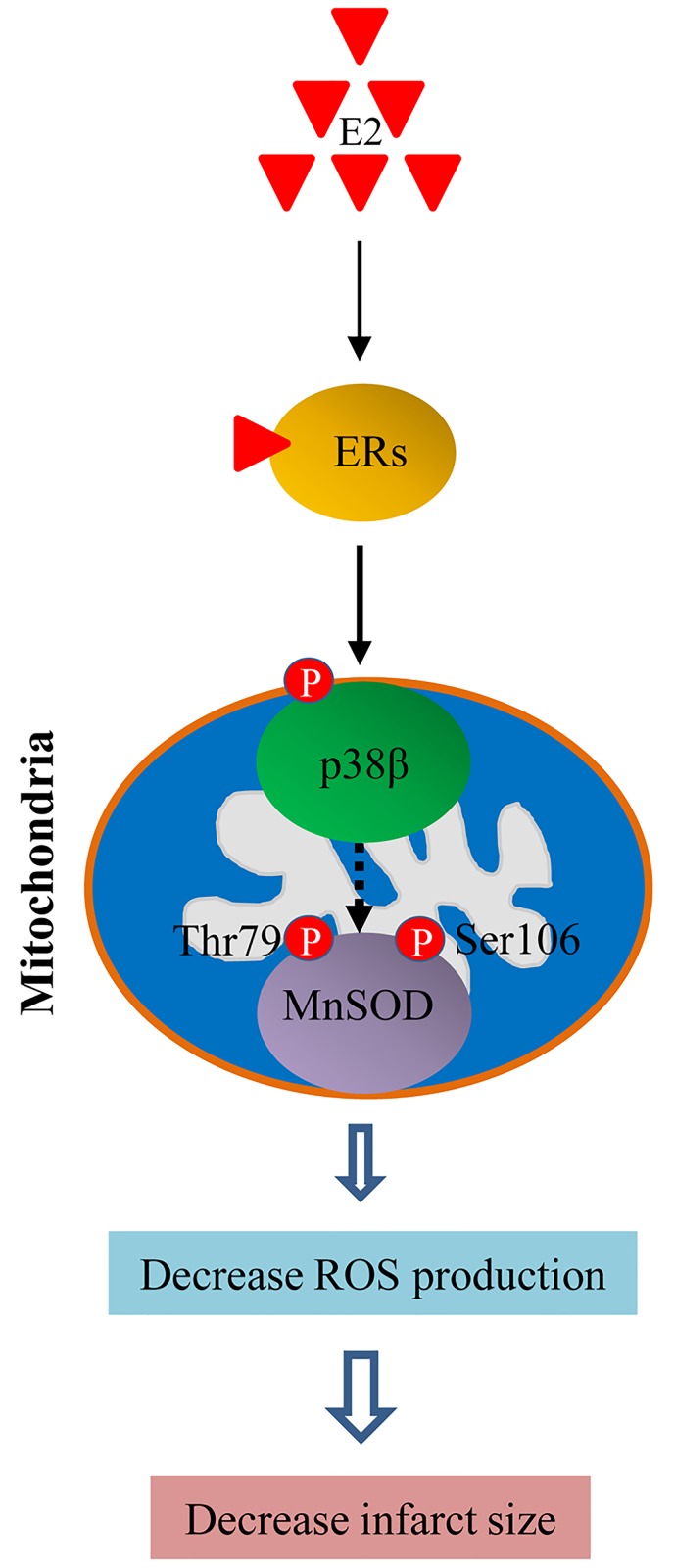
A working mechanism representing the E2/ER-mediated cardioprotection from I/R injury via p38β and MnSOD. In cardiomyocytes, E2, via ERα and ERβ, increases mitochondrial p38β activation. Phosphorylated p38β (activated p38β kinase), enhances MnSOD activity through phosphorylating the dismutase at T79 and S106 residue, leading to suppression of intracellular ROS production and decreases the myocardial injury and infarct size during I/R.

In this report, we expand our previous observations to an in-vivo, whole animal model to show that E2, via both ERα and ERβ, protects the heart from I/R injury by increasing the activity of mitochondrial p38β and MnSOD in the female murine heart. Furthermore, our data shed light on the molecular mechanisms behind the p38β –MnSOD interaction, demonstrating for the first time to our knowledge that threonine 79 (T79) and serine 106 (S106) of MnSOD are the phosphorylation sites for the kinase, and that the mutation of these two residues negate the key function of MnSOD against H/R. A schematic diagram summarizing the major findings is pictured in Fig 7.

MnSOD is a major mitochondrial ROS scavenging enzyme indispensable to the cellular defense system against oxidative stress created during I/R injury. It exists in homotetramers and localizes to the mitochondrial matrix. Post-translational modification of superoxide dismutases is an important part of the SOD regulation and can alter the enzyme function against oxidative stress [33]. In non-cardiac cells, S106 residue of MnSOD was found to be phosphorylated by the mitochondrial cell cycle-dependent kinase 1(Cdk1) and Cdk4, leading to enhanced MnSOD activity, as part of a pro-survival mechanism during genotoxic stress of irradiation [34, 35]. While E2 has been implicated in the modulation of MnSOD in other tissues, the role of estrogen in support of MnSOD function in cardiomyocytes has not been well characterized [36–38]. We have previously reported that E2-activated p38β significantly reduces mitochondrial ROS after H/R and that a mitochondrial pool of p38β exists that interacts with MnSOD in NRCM [16, 17]. p38β belongs to the p38 MAPK family, which includes p38α, p38β, p38γ, and p38δ [39]. Of the four, the first two are expressed predominantly in the heart [40]. The p38β knockout mice express cardiac structural abnormality, and murine hearts expressing p38β dominant negative mutants have poor functional recovery after I/R [41, 42]. In cardiomyocytes, p38β has been linked to antioxidative, prosurvival processes and cardiac hypertrophy, although the mechanism behind the p38β-associated suppression of ROS was unclear [16, 43].

In the present study, we provide a novel working mechanism in which E2/ER upregulates mitochondrial p38β activity, with subsequent phosphorylation of the dismutase at S106 and T79 by p38β, leading to enhanced SOD activity, suppressed ROS and reduced myocardial infarct post I/R. The findings of this study add to the current understanding of the role E2 plays in redox regulation, and shed light on the molecular mechanisms behind the actions of estrogen in protecting the ischemic heart.

## Supporting Information

S1 Fig Genotyping of WT mice and ER null mice. The WT female mice express both ERα gene (741bp) and ERβ gene (1001bp). ERKO has Neo disruption of the ERα gene (223-bp) and intact ERβ (1001bp). BERKO contains the intact ERα gene (741-bp) and Neo disruption of the ERβ gene (593-bp). DERKO contains both Neo disruption of both the ERα gene (223-bp) and ERβ (593-bp).
(TIF)

S2 Fig Sequences of WT and mutant MnSOD peptides used for kinase activity assay. A. Four sequences of WT MnSOD peptides (MnSOD-1, MnSOD-2, MnSOD-3, and MnSOD-4) with high surface probability and high score of phosphorylation by MAPK are underlined and in bold. B. Two WT MnSOD peptides (MnSOD-2 and MnSOD-3, underlined and in bold) identified to be phosphorylated and 5 mutant derivative peptides (MnSOD-2-1, MnSOD-2-2, MnSOD-3-1, MnSOD-3-2, and MnSOD-3-3) containing a point mutation of each threonine (t) and serine (s) residues to alanine (a) within MnSOD-2 or MnSOD-3.
(TIF)

S3 Fig The plasmid map of the pcDNA3.1/ (WT) MnSOD 3’-flags and pcDNA3.1/ (Mut) MnSOD 3’-flags. The plasmids, the pcDNA3.1/ (WT) MnSOD 3’-flags and pcDNA3.1/ (Mut) MnSOD 3’-flags, are gifts from Dr. Jianjian Li at the University of California, Davis, and their construction was detailed previously [35]. T79A MnSOD mutation was generated from the pcDNA3.1/ (WT) MnSOD 3’-flags using QuikChange^®^ site-directed mutagenesis kit by changing the 79th residue from ACA (Threonine) to GCA (Alanine). The mutation PCR primers are as follows. hMnSOD 79T-A F: 5’-GCC AAG GGA GAT GTT GCA GCC CAG ATA GCT C-3’; hMnSOD 79T-A R: 5’-G AGC TAT CTG GGC TGC AAC ATC TCC CTT GGC-3’.
(TIF)

S4 Fig Sequencing result of the full length MnSOD T79A mutant. Upper (Query) is the sequence of wild type MnSOD and lower (Sbjct) is the mutated MnSOD, illustrating in the highlighted lineup the nucleotide A in the Query mutated to G in the Sbjct, changing the amino acid residue 79 from Threonine (ACA) to Alanine (GCA).
(TIF)

S5 Fig Transfection rate of WT MnSOD plasmid and mutant MnSOD plasmids in primary culture of neonatal rat cardiomyocytes. DAPI (blue) was used to stain the nucleus, and antibody to the DYKDDDDK epitope, DyLight 680 conjugate (FG4R), was used to identify FLAG sequence of the expressed plasmids (red) in transfected NRCM. Scale bar = 25 μm. DAPI, 4’,6-diamidino-2-phenylindole; MnSOD, manganese superoxide dismutase; pcDNA 3.1/ (WT) MnSOD, wild type full length MnSOD plasmid; pcDNA 3.1/ (T79A) MnSOD, full length MnSOD plasmid containing point mutation of threonine 79 changed to alanine; pcDNA 3.1/ (S106A) MnSOD, full length MnSOD plasmid containing point mutation of serine 106 changed to alanine.
(TIF)

S1 Table Sequences of WT MnSOD peptides used for kinase assay. (TIF)

S2 Table Sequences of WT and mutant MnSOD peptides used for kinase assay. (TIF)
