# Supplementary figures and images for: Estrogen Protects the Female Heart from Ischemia/Reperfusion Injury through Manganese Superoxide Dismutase Phosphorylation by Mitochondrial p38β at Threonine 79 and Serine 106
Source: PLoS One. 2016 Dec 8;11(12):e0167761. doi: 10.1371/journal.pone.0167761 (PMC5145184; doi:10.1371/journal.pone.0167761)

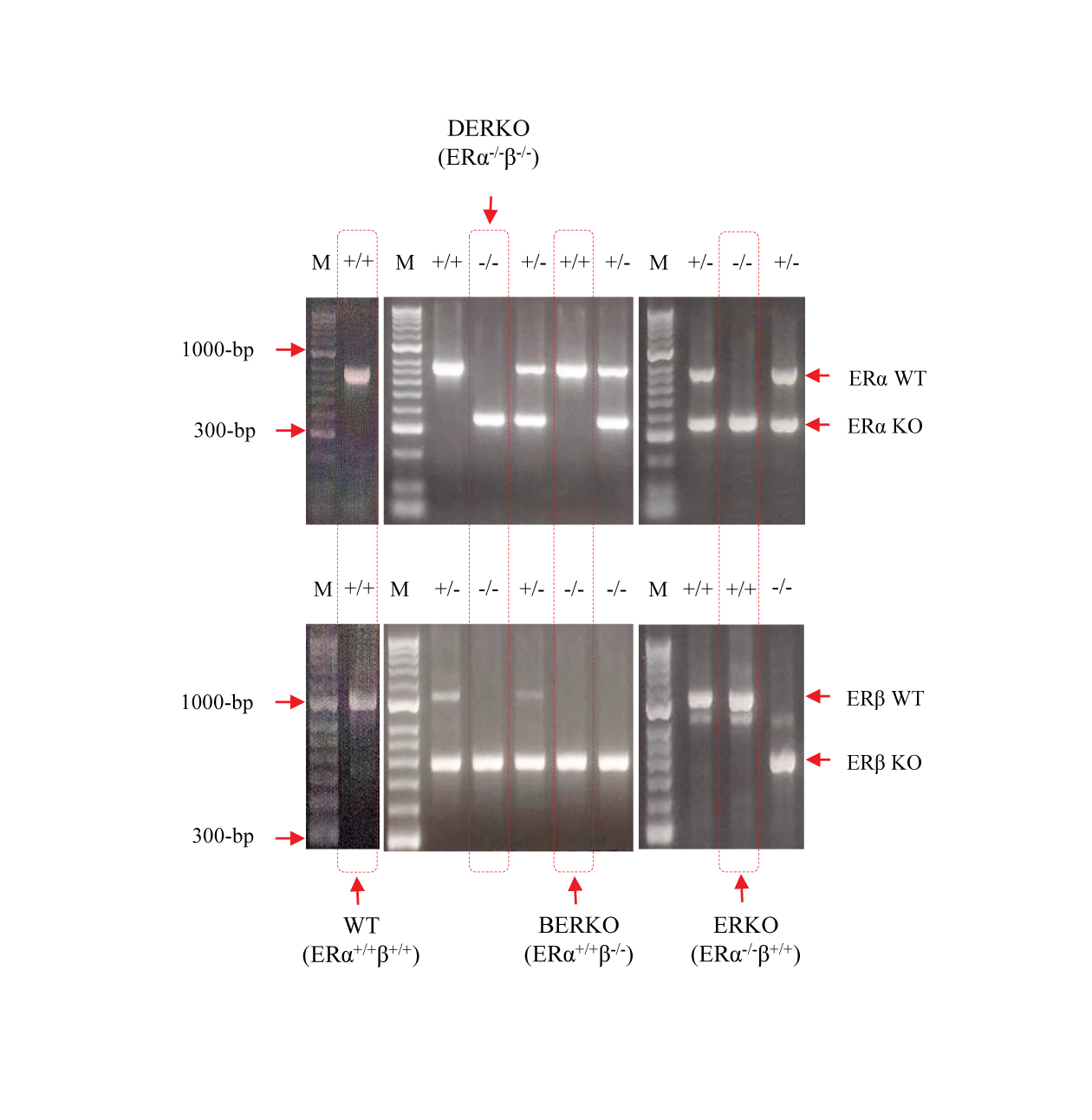

Supplement: S1 Fig — The WT female mice express both ERα gene (741bp) and ERβ gene (1001bp). ERKO has Neo disruption of the ERα gene (223-bp) and intact ERβ (1001bp). BERKO contains the intact ERα gene (741-bp) and Neo disruption of the ERβ gene (593-bp). DERKO contains both Neo disruption of both the ERα gene (223-bp) and ERβ (593-bp). (TIF) [file pone.0167761.s001.tif]

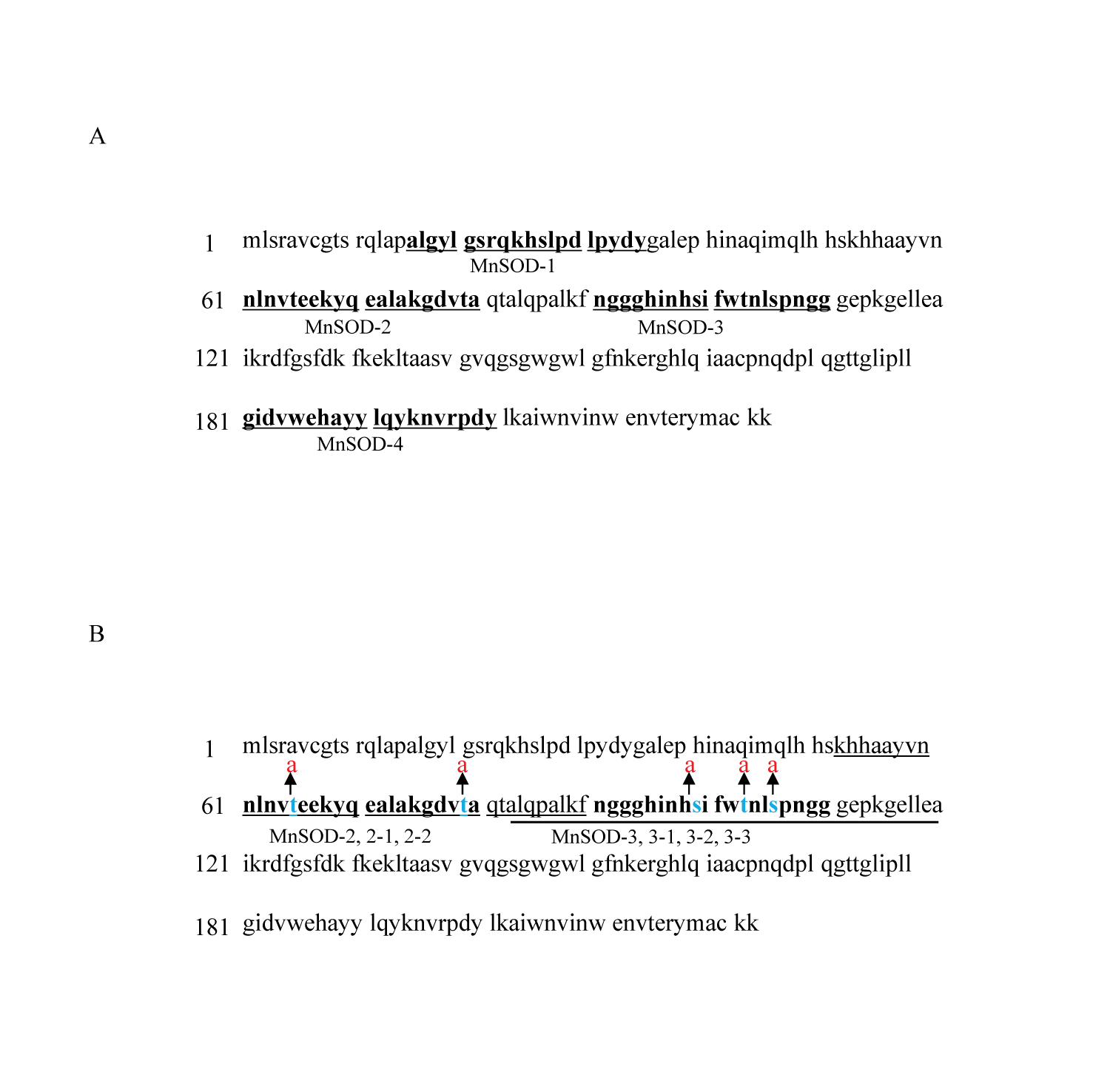

Supplement: S2 Fig — A. Four sequences of WT MnSOD peptides (MnSOD-1, MnSOD-2, MnSOD-3, and MnSOD-4) with high surface probability and high score of phosphorylation by MAPK are underlined and in bold. B. Two WT MnSOD peptides (MnSOD-2 and MnSOD-3, underlined and in bold) identified to be phosphorylated and 5 mutant derivative peptides (MnSOD-2-1, MnSOD-2-2, MnSOD-3-1, MnSOD-3-2, and MnSOD-3-3) containing a point mutation of each threonine (t) and serine (s) residues to alanine (a) within MnSOD-2 or MnSOD-3. (TIF) [file pone.0167761.s002.tif]

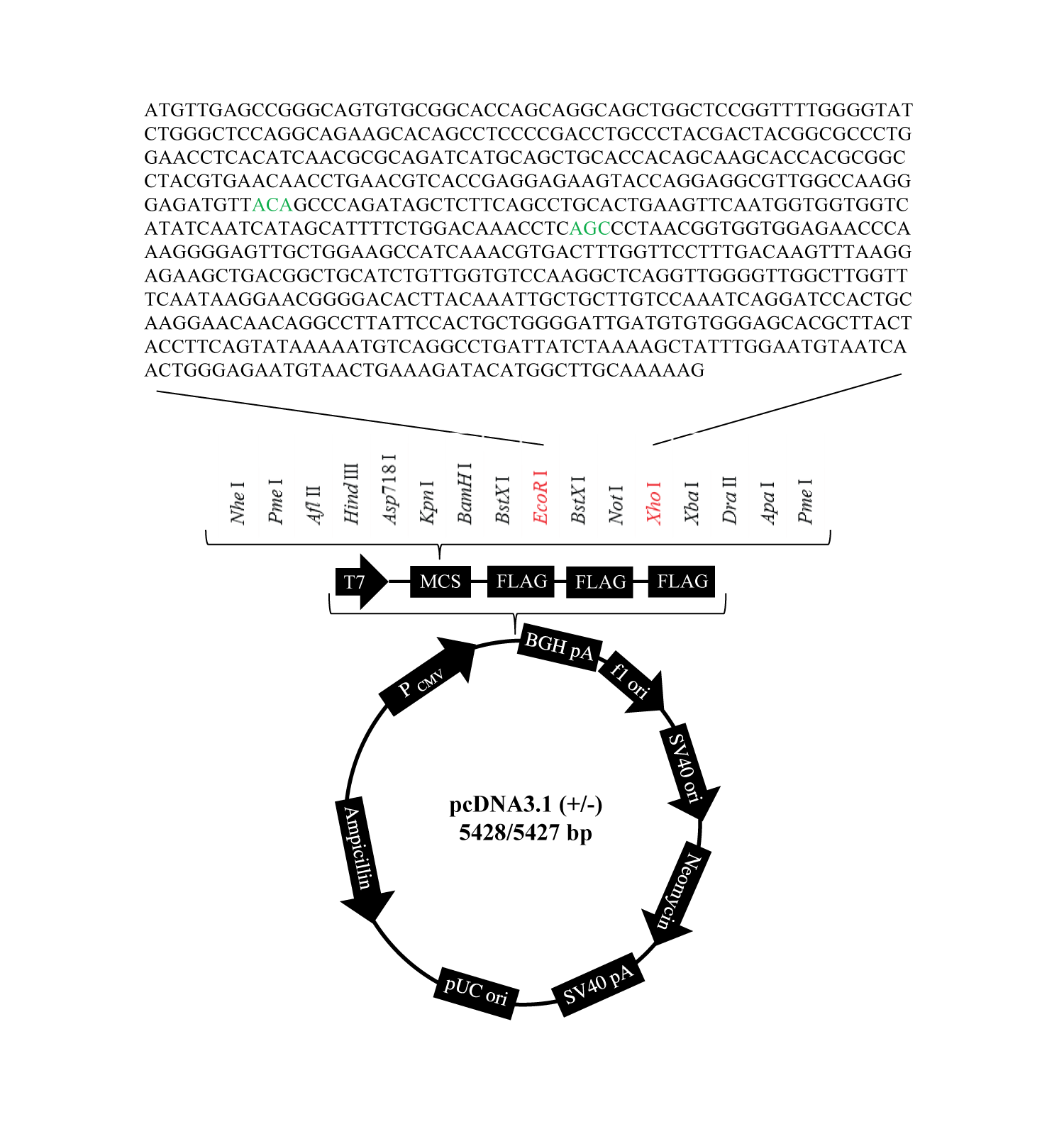

Supplement: S3 Fig — The plasmids, the pcDNA3.1/ (WT) MnSOD 3’-flags and pcDNA3.1/ (Mut) MnSOD 3’-flags, are gifts from Dr. Jianjian Li at the University of California, Davis, and their construction was detailed previously [35]. T79A MnSOD mutation was generated from the pcDNA3.1/ (WT) MnSOD 3’-flags using QuikChange® site-directed mutagenesis kit by changing the 79th residue from ACA (Threonine) to GCA (Alanine). The mutation PCR primers are as follows. hMnSOD 79T-A F: 5’-GCC AAG GGA GAT GTT GCA GCC CAG ATA GCT C-3’; hMnSOD 79T-A R: 5’-G AGC TAT CTG GGC TGC AAC ATC TCC CTT GGC-3’. (TIF) [file pone.0167761.s003.tif]

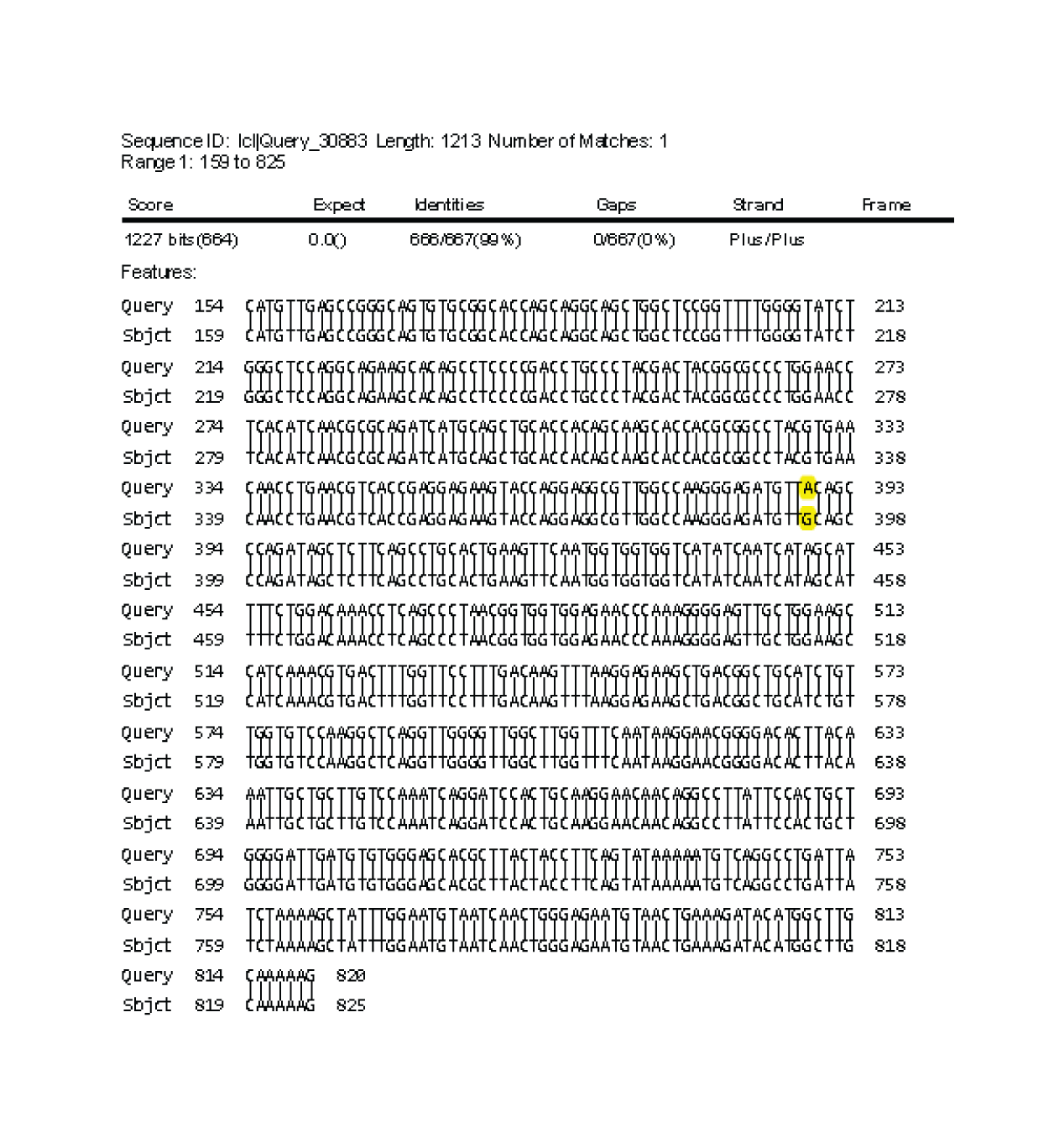

Supplement: S4 Fig — Upper (Query) is the sequence of wild type MnSOD and lower (Sbjct) is the mutated MnSOD, illustrating in the highlighted lineup the nucleotide A in the Query mutated to G in the Sbjct, changing the amino acid residue 79 from Threonine (ACA) to Alanine (GCA). (TIF) [file pone.0167761.s004.tif]

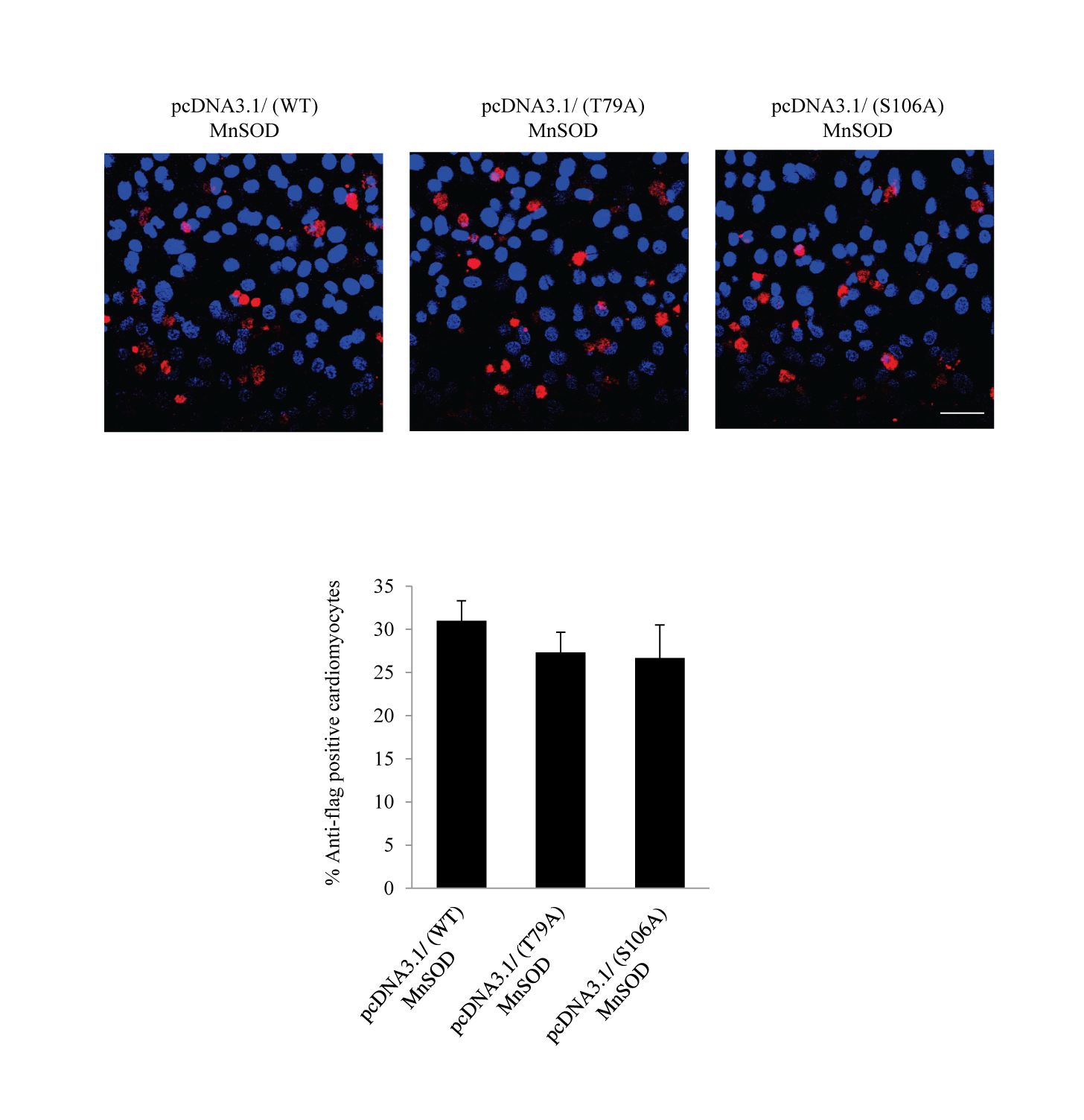

Supplement: S5 Fig — DAPI (blue) was used to stain the nucleus, and antibody to the DYKDDDDK epitope, DyLight 680 conjugate (FG4R), was used to identify FLAG sequence of the expressed plasmids (red) in transfected NRCM. Scale bar = 25 μm. DAPI, 4’,6-diamidino-2-phenylindole; MnSOD, manganese superoxide dismutase; pcDNA 3.1/ (WT) MnSOD, wild type full length MnSOD plasmid; pcDNA 3.1/ (T79A) MnSOD, full length MnSOD plasmid containing point mutation of threonine 79 changed to alanine; pcDNA 3.1/ (S106A) MnSOD, full length MnSOD plasmid containing point mutation of serine 106 changed to alanine. (TIF) [file pone.0167761.s005.tif]

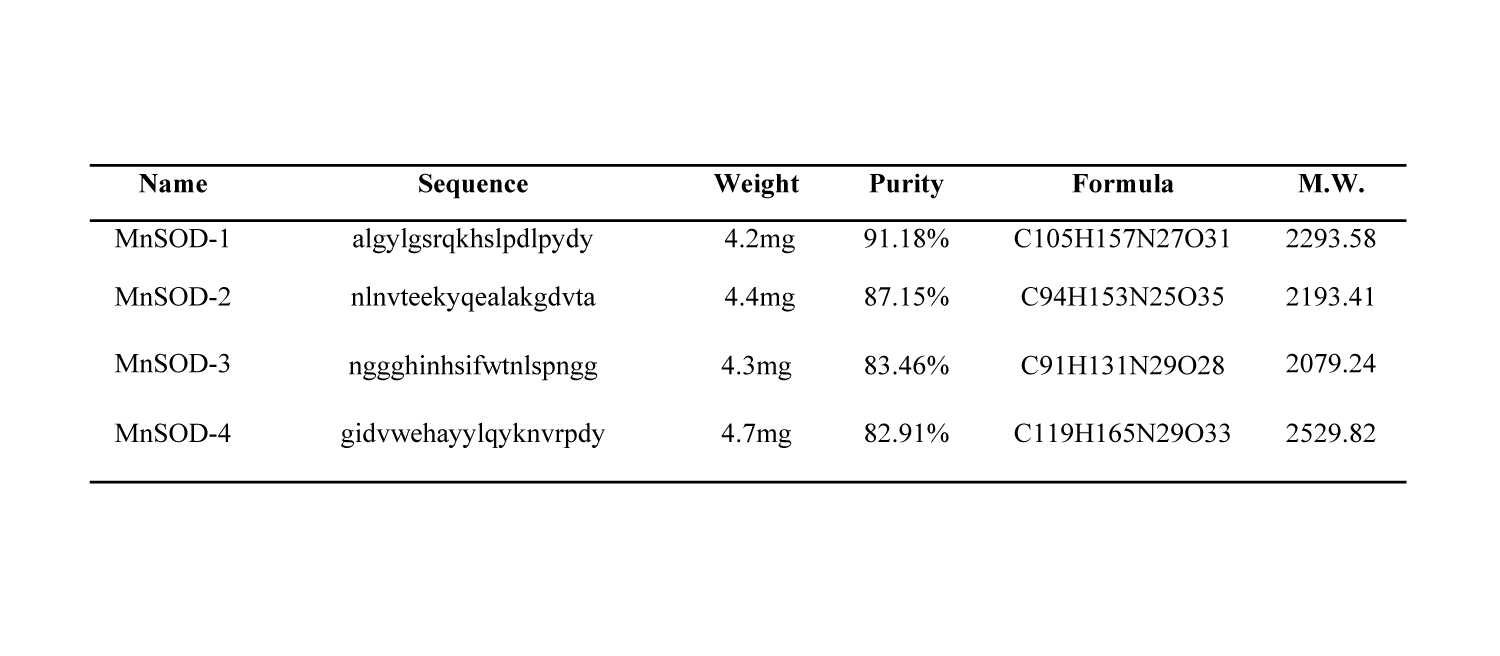

Supplement: S1 Table — (TIF) [file pone.0167761.s006.tif]

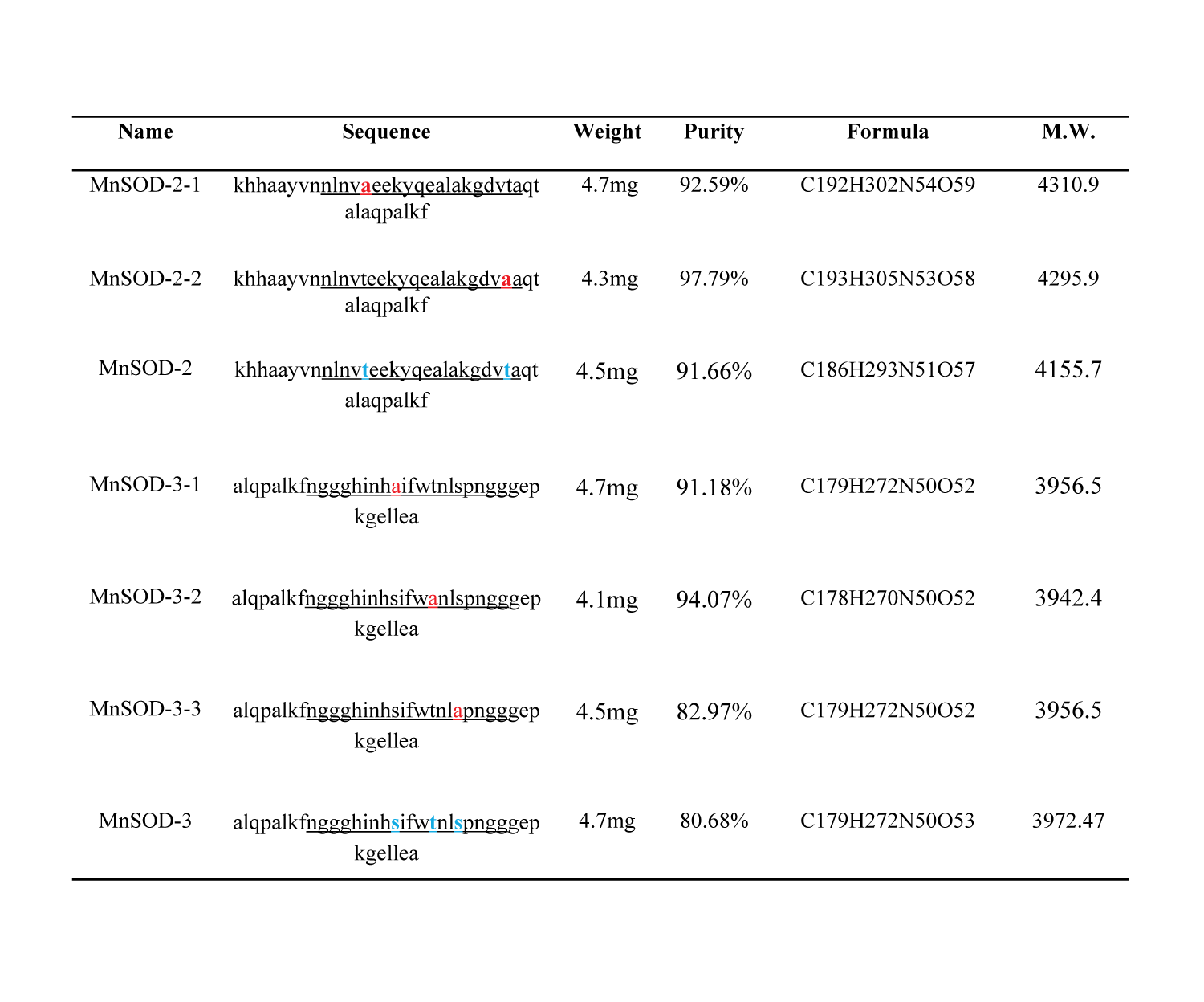

Supplement: S2 Table — (TIF) [file pone.0167761.s007.tif]
